# A215 EVALUATING THE COMPARABILITY OF CARE FOR PERSONS ADMITTED TO TORONTO AREA HOSPITALS WITH ACUTE SEVERE ULCERATIVE COLITIS

**DOI:** 10.1093/jcag/gwac036.215

**Published:** 2023-03-07

**Authors:** S Vukovic, X You, S Roberts, F Razak, A Verma, L Targownik

**Affiliations:** 1 Internal Medicine, University of Toronto; 2 Internal Medicine, St. Michael's-Unity Health; 3 Gastroenterology, Mount Sinai Hospital, Toronto, Canada

## Abstract

**Background:**

Approximately 20% of patients with ulcerative colitis will experience an acute severe exacerbation requiring hospitalization. Acute severe ulcerative colitis (ASUC) is a medical emergency associated with significant morbidity and a mortality rate of 1%. Timely initiation of treatment and assessment of clinical response is critical in the management of ASUC. With an aim to reduce treatment variability and improve outcomes, multiple gastrointestinal societies have published guidelines highlighting recommendations for optimal care in ASUC. It remains unclear how closely these guidelines are implemented in clinical practice. Measuring adherence to these recommended processes of care may act as a surrogate measure for quality of care and a way to indirectly evaluate outcomes in the management of patients with ASUC. Studies have shown that even amongst experienced providers practice pattern variability exists. Identifying significant variations in the management of patients with ASUC will highlight where improvement in guideline dissemination and greater adherence is required.

**Purpose:**

We sought to evaluate how quality of care indicators varied across 7 hospital sites for patients admitted ASUC in the Greater Toronto Area.

**Method:**

Using GEMINI, a research collaborative that collects and analyses data from inpatient admissions at 7 Toronto area hospitals, we identified patients admitted to hospital with ASUC from June 2016-December 2019. Hospital sites were further categorized into 3 hospital types; 1 IBD specialty centre (ISC), 3 other academic centres (AC) and 3 community centres (CC). Process measures assessed included proportion tested for C-reactive protein at baseline and following treatment initiation, duration of corticosteroid use, timing and initiation of biologic agents, rates of venous thromboembolism prophylaxis and opioid use. Outcome measures included hospital length of stay, rates of colectomy and mortality.

**Result(s):**

765 hospitalizations were included in the study; 320 occurring at ISC, 308 at AC and 137 at CC. Corticosteroid use on admission were highest at the ISC at 78% compared to 64% at AC and 63% at CC (p <0.001). Among those who received steroids on admission, 47% of patients remained on intravenous corticosteroids for at least 5 days in the ISC compared to 39% in AC and 75% in CC (p< 0.001). Initiation of biologic rescue therapy was highest at the ISC occurring in 37% of hospitalizations compared to 22% in AC and 23% in CC (p<0.001). In addition, VTE prophylaxis rates were highest at the ISC at 83% followed by 60% in AC and 45% in CC (p<0.001). Rates of colectomy were highest at ISC (12% of hospitalizations vs. 7% in AC).

**Conclusion(s):**

Greater adherence to indicators of quality of care were seen at the ISC compared to ACs and CCs, although patient outcomes assessed were not clearly different between sites. Further strategies are required to improve adherence to markers of quality care for patients admitted with ASUC.

**Please acknowledge all funding agencies by checking the applicable boxes below:**

None

**Disclosure of Interest:**

None Declared

